# Sex-Specific Diurnal Immobility Induced by Forced Swim Test in Wild Type and Clock Gene Deficient Mice

**DOI:** 10.3390/ijms16046831

**Published:** 2015-03-25

**Authors:** Ningyue Li, Yanhua Xu, Xiaojuan Chen, Qing Duan, Mei Zhao

**Affiliations:** Key Lab of Mental Health, Institute of Psychology, Chinese Academy of Sciences, Beijing 100101, China; E-Mails: yueyue1989825@sohu.com (N.L.); xuyanhua.stu@hotmail.com (Y.X.); cxj2013@gmail.com (X.C.); duanq@psych.ac.cn (Q.D.)

**Keywords:** circadian, clock gene, mutant, sex-difference, immobility, forced swim

## Abstract

Objective: The link between alterations in circadian rhythms and depression are well established, but the underlying mechanisms are far less elucidated. We investigated the circadian characteristics of immobility behavior in wild type (WT) mice and mice with mutations in core *Clock* genes. Methods: All mice were tested with forced swim test (FST) at 4 h intervals. Results: These experiments revealed significant diurnal rhythms associated with immobility behavior in both male and female WT mice with sex-different circadian properties. In addition, male mice showed significantly less immobility during the night phase in comparison to female mice. Female *Per1^Brdm1^* mice also showed significant rhythmicity. However, the timing of rhythmicity was very different from that observed in female wild type mice. Male *Per1^Brdm1^* mice showed a pattern of rhythmicity similar to that of wild type mice. Furthermore, female *Per1^Brdm1^* mice showed higher duration of immobility in comparison to male *Per1^Brdm1^* mice in both daytime and early night phases. Neither *Per2^Brdm1^* nor *Clock^Δ19^* mice showed significant rhythmicity, but both female *Per2^Brdm1^* and *Clock^Δ19^* mice had lower levels of immobility, compared to males. Conclusions: This study highlights the differences in the circadian characteristics of immobility induced by FST in WT, *Clock^Δ19^*, *Per1*, and *Per2* deficient mice.

## 1. Introduction

Organisms live under the strong influence of day-night cycles, which has resulted in the development of a highly conserved circadian clock system that allows them to adjust their activities in response to recurring environmental changes. Increasing evidence suggests that alterations in circadian rhythms can have profound consequences on emotional behavior and mental health [1]. Both circadian and infradian rhythms affect human moods [2]. Patients affected by a major depressive episode often report diurnal variations of mood with typical mood worsening in the morning [3]. In addition, affective disorders, such as major depressive disorder (MDD), bipolar disorder (BP), and seasonal affective disorder (SAD) are associated with major disruptions in circadian rhythms [4].

Circadian rhythms are endogenously driven biological variations that fluctuate with a periodicity of approximately 24 h. The master circadian pacemaker, located in the hypothalamic suprachiasmatic nucleus (SCN), controls many physiological and behavioral variables. This core molecular clock is composed of a series of transcriptional and translational feedback loops that cycle over 24 h. Briefly, the CLOCK and BMAL1 heterodimerize and bind to *cis*-regulatory elements in a number of genes, including the three *Period* genes (*Per1*, *Per2* and *Per3*) and the two *Cryptochrome* (*Cry1* and *Cry2*) genes, and drive their expression. In turn, the expressed period and cryptochrome proteins suppress the activity of CLOCK and BMAL1 [5].

Abnormalities in the core circadian genes have been implicated in mood disorders [6]. Human genetic studies associate polymorphic variations of the *Clock*, and *Clock*-related genes, with MDD, BP and SAD [7,8]. The *Clock*^Δ19^ mutant mice display hyperactivity, less depression-like behavior, less anxiety, and increased cocaine reward values—all traits that are similar to human mania [9]. *Per2* mutant mice (*Per2^Brdm1^*) have reduced immobility in the forced swim test (FST), while mice with mutations in both *mPer1* and *mPer2 (mPer1*/*mPer2^ldc^*) have increased anxiety-related behavior [10,11]. Although these studies have indicated that the genes involved in the central circadian rhythm may regulate mood-related behaviors, few studies have investigated their roles in the circadian changes of those behaviors.

FST is now widely used both in basic research and in pharmaceutical screens for potential antidepressant treatments due to its high predictive validity [12]. In addition, “behavioral despair” just as increased immobility and decreased swimming or climbing in FST is, by some, considered an indication of “depressive-like” symptomatology [13]. To investigate the roles of core clock genes in the rhythm of response to FST, we characterized the circadian patterns of immobility behavior in wild type, *Clock^Δ19^*, *Per1* (*Per1^Brdm1^*) and *Per2* (*Per2^Brdm1^*) mutant mice at 4 h intervals. Sex-specific differences in the timing of the circadian system are important in determining responses to both endogenous and exogenous factors. Importantly, depression is 1.5–3 times more prevalent among women [14], indicating a sex-specific difference in depression. However, only few studies have investigated sex-specific differences in relation to the effect of circadian rhythm disturbance in depression. To address this issue, both male and female wild type (WT) and mutant mice were included in this study.

## 2. Results and Discussion

### 2.1. Results

#### 2.1.1. Sex-Specific Circadian Rhythms in the Duration of Immobility in Wild Type Mice

Table 1 shows significant daily rhythms of swimming activity in WT (*F* = 5.24, *p* < 0.05 for males and *F* = 11.39, *p* < 0.05 for females). The properties of the rhythms were sex-specific in WT animals. For WT male mice, the peak time was advanced by approximately 5 h (*p* = 0.016), while the nadir was advanced by approximately 9 h (*p* = 0.003) in comparison to female mice.

#### 2.1.2. Circadian Rhythms in the Duration of Immobility Were Changed in Female, but not Male *Per1^Brdm1^* Mice

Table 1 also shows significant daily rhythms of immobility in *Per1^Brdm1^* mice (*F* = 7.35, *p* < 0.05 for males and *F* = 7.82, *p* < 0.05 for females). However, there was no significant difference in the peak or nadir times between male and female *Per1^Brdm1^* mice. There were also no differences when the peak and nadir times of male *Per1^Brdm1^* mice were compared to WT mice. In contrast, the peak and nadir times of female *Per1^Brdm1^* mice were advanced by approximately 10 h (*p* < 0.001 for both peak and nadir times compare to WT mice).

**Table 1:** Circadian properties in the duration of immobility in wild type and *Per1^Brdm1^*.

Parameter	Type of Mice			
	Wild Type		*Per1^Brdm1^*	
	Male	Female	Male	Female
Mesor	167.19 ± 5.66	169.88 ± 12.46	154.42 ± 10.29	177.55 ± 11.73
Amplitude	40.33 ± 6.56	29.59 ± 1.88	27.36 ± 5.39	35.91 ± 2.58
Peak time	10.08 ± 1.43	15.17 ± 0.36 ^#^	6.22 ± 2.55	5.33 ± 0.24
Nadir time	18.42 ± 2.08	3.17 ± 0.36 ^#^	15.22 ± 0.46	17.33 ± 0.24
Cosinor *p* value	*F* = 5.24 *	*F* = 11.39 *	*F* = 7.35 *	*F* = 7.82 *

Mesor = the middle value of the fitted cosine that represents a rhythm-adjusted mean; Amplitude = half of the difference between the minimum and maximum of the fitted cosine functions; ^#^ *p* < 0.05 peak time and nadir time compared to male mice of the same genotype; * *p* < 0.05, amplitude (*F*-test).

#### 2.1.3. *Per2^Brdm1^* and *Clock^Δ19^* Mice Lost the Circadian Rhythm in Their Duration of Immobility

Table 2 shows that neither male nor female mice demonstrated significant daily rhythms of immobility, neither in *Per2^Brdm1^* nor *Clock^Δ19^* mice (Table 2).

**Table 2:** Circadian properties of the duration of immobility in *Per2^Brdm1^* and *Clock^Δ19^*.

Parameter	Type of Mice			
	*Per2^Brdm1^*		Clock^Δ19^	
	Male	Female	Male	Female
Mesor	195.89 ± 10.42	175.21 ± 5.07	174.36 ± 8.65	125.35 ± 5.88
Amplitude	30.26 ± 5.41	25.18 ± 3.62	59.53 ± 5.21	28.86 ± 5.71
Cosinor *p* value	*F* = 0.06	*F* = 2.26	*F* = 0.63	*F* = 0.66

Mesor = the middle value of the fitted cosine that represents a rhythm-adjusted mean; Amplitude = half of the difference between the minimum and maximum of the fitted cosine functions.

#### 2.1.4. Sex-Specific Differences in the Duration of Immobility within Each Genotype of Mice

**Figure 1:**
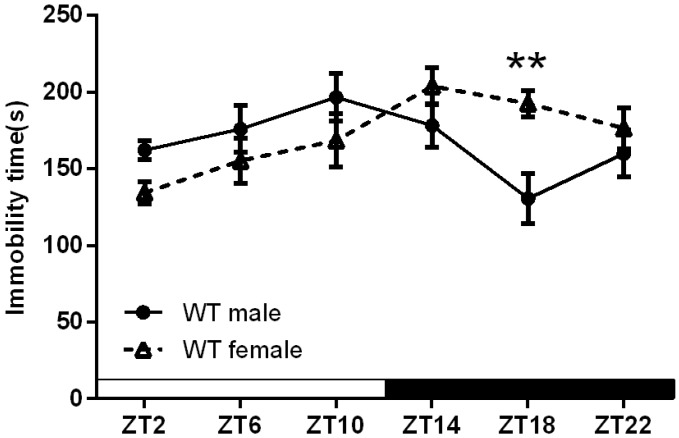
Sex-specific effects on the duration of immobility in wild type mice. The data are expressed as the mean ± SEM. *n* = 7 mice for each group. ** *p* < 0.01.

Figure 1 shows sex-specific differences in the duration of immobility in WT mice. Two way ANOVA showed significant effects on sex (*F*_(1.72)_ = 2.538, *p* < 0.05) and the interaction between sex and Zeitgeber effect (*F*_(1.72)_ = 3.607, *p* < 0.05). Male and female mice showed very similar durations of immobility in the day phase, while male mice showed a significantly lower duration of immobility in the night phase compared to female mice (*p* < 0.01 at ZT18).

**Figure 2:**
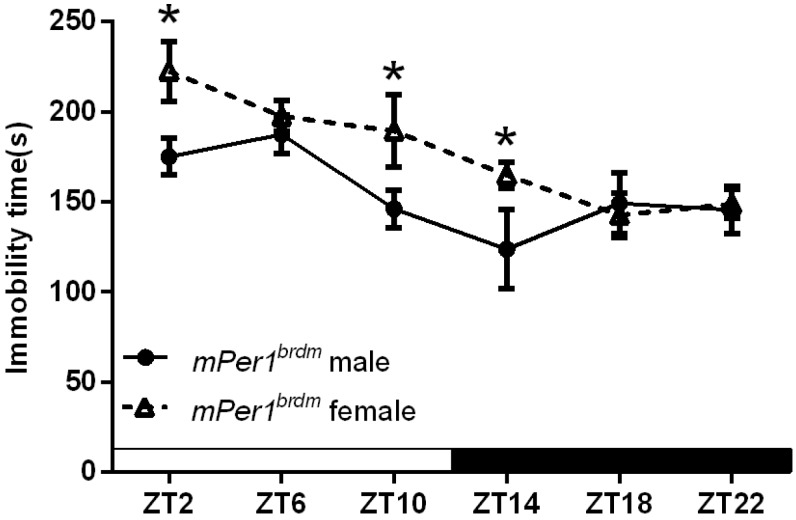
Sex-specific effects on the duration of immobility in *Per1^Brdm1^* mice. The data are expressed as the mean ± SEM. *n* = 8 and 9 mice for male and female, respectively. * *p* < 0.05.

Figure 2 shows sex-specific differences in the duration of immobility in *Per1^Brdm1^* mice. Two way ANOVA showed that there were significant sex-specific (*F*_(1.90)_ = 8.431, *p* < 0.01) and Zeitgeber (*F*_(5.90)_ = 6.327, *p* < 0.001) effects, but no significant interaction of the two factors. The duration of immobility of male *Per1^Brdm1^* mice was significantly lower than that of female mice at ZT2, ZT10, and ZT14 (*p* < 0.05).

**Figure 3:**
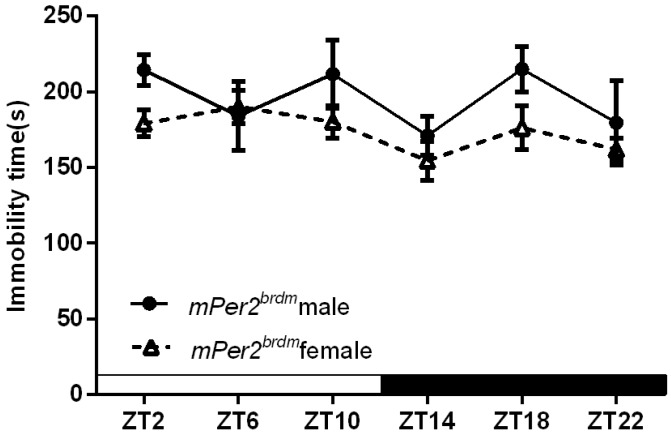
Sex-specific effects on the duration of immobility in *Per2^Brdm1^* mice. The data are expressed as the mean ± SEM. *n* = 6 and 8 mice for male and female, respectively.

Figure 3 shows sex-specific differences in the duration of immobility in *Per2^Brdm1^* mice. Two way ANOVA showed that there was a significant sex-specific effect (*F*_(1.72)_ = 6.746, *p* < 0.05), but no significant differences in the Zeitgeber effect or in its interaction with sex. *Post hoc* tests showed trends at ZT2 and ZT18 (*p* = 0.06 and 0.09, respectively).

**Figure 4:**
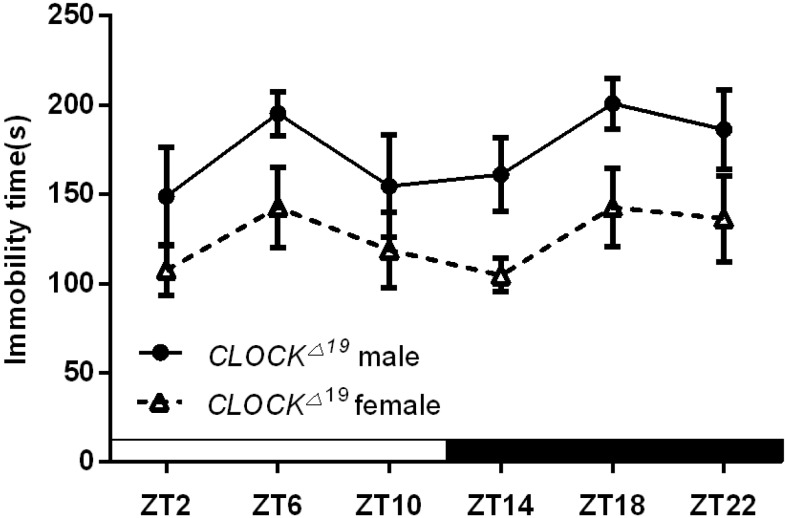
Sex-specific effects on the duration of immobility in *Clock^Δ19^* mice. The data are expressed as the mean ± SEM. *n* = 7 mice for each group.

Figure 4 shows sex-specific differences in the duration of immobility in *Clock^Δ19^* mice. Two way ANOVA showed that there was a significance in sex-specific effect (*F*_(1.72)_ = 16.74, *p* < 0.001) but none in Zeitgeber effect nor in its interaction with sex. *Post hoc* tests showed trends at ZT6, ZT14, ZT18 and ZT22 (*p* = 0.07, 0.058, 0.051 and 0.09, respectively).

### 2.2. Discussion

This study identified diurnal rhythms of immobility induced by FST in WT mice. Male WT mice reached their peak near late daytime (ZT10) with a nadir near midnight (ZT18), while female wild types reached their peak near early night (ZT14) with a nadir near early daytime (ZT2). Female WT mice showed characteristics very similar to those of negative mood in human studies, where peak depression also occurred in the early active period [2]. However, due to the sample size, the character of participants, and the data collection methods, studies investigating a link between circadian rhythms and human mood have produced mixed results. In a study of working females, peaks for positive emotions were detected at noon and again in the evenings, while peaks in negative emotions were found at mid-morning and mid-afternoon [15]. In a study of college students, the only peak for positive emotions occurred in the evening, while the only peak for negative emotions occurred in the early morning [2]. In the present study, we profiled circadian rhythms of immobility in FST in both male and female mice. It is hypothesized that circadian clock dysfunction, which is primarily regulated by clock genes, leads to mood disorders [1,4]. Here, we further investigated the role of core circadian genes in the regulation of circadian rhythms of immobility response in the FST.

We found that both male and female *Clock* mutant mice lost their diurnal rhythms of immobility in FST, indicating its potential role in maintaining the circadian rhythms of depression-like behavior. There are a few studies revealing the possible roles of the *Clock* gene in mood disorders. For example, chronic treatment with the antidepressant fluoxetine increases the expression of *Clock* in the hippocampus [16], while disruption of *Clock* expression induces mania-like behavior [9]. Human studies found that the *Clock* gene with a polymorphism in the 3'-flanking region has been associated with greater severity of insomnia during antidepressant treatment [17] and a higher recurrence rate of bipolar episodes [18]. However, Benedetti *et al.* failed to find a possible effect of the *Clock* genotype on the regulation of perceived diurnal mood fluctuations during a major depressive episode [18]. This observation is not implicitly conflicting with our findings for two reasons: firstly, the *Clock* mutant mice used in our study possesses a deletion in exon 19 of the *Clock* gene which is different from the human genotype; secondly, “behavioral despair” induced by FST in mice can’t entirely mimic the mood of depressive episode in humans.

We also found that mice that are homozygous for the targeted allele of either *mPer1* or *mPer2* showed distinct circadian changes of immobility induced by FST in comparison to WT mice. *Per1^Brdm1^* female mice showed significant diurnal rhythms of immobility behavior with delays in both peak and nadir times when compared to female WT mice. The pattern of diurnal rhythms of immobility for male *Per1^Brdm1^* mice was very similar to that of WT male mice. In contrast, neither male nor female *Per2^Brdm1^* mice showed significant rhythmicity. These results indicate that *mPer1* and *mPer2* play important but distinct roles in the regulation of circadian rhythms in depression-like behavior. These genes also have distinct roles in circadian clock function [19]. In addition, the circadian rhythm of circulating glucocorticoids differs between *Per1^Brdm1^* and *Per2^Brdm1^* mice: *Per1^Brdm1^* mice show markedly elevated levels of circulating glucocorticoids lacking any circadian rhythm, whereas *Per2^Brdm1^* mice demonstrate elevated but diurnally fluctuating serum glucocorticoids levels [20]. Dysregulation in either the CLOCK system or the hypothalamic-pituitary-adrenal (HPA) axis may cause similar, pathologic manifestations by uncoupling circulating cortisol concentrations from tissue sensitivity to glucocorticoids [21]. Therefore, further studies should investigate whether the complex crosstalk between the circadian CLOCK system and the HPA axis are involved in the effects of the *Clock* genes on depression.

Anxiety and depression occur more frequently in females than in males in the human population [22], indicating the existence of sex-specific differences in the mechanisms of depression. Most importantly, sex hormones affect many aspects of circadian responses, and there are significant sex-specific differences in rhythmicity [23]. Here, we show that WT mice display diurnal rhythms of immobility with a sex-specific pattern. This implies that male–female differences also exist in the interaction of environmental factors and internal circadian rhythmicity. Furthermore, *Per1^Brdm1^* mice showed circadian rhythms of immobility, but no sex-specific differences were observed. Our results implied that *mPer1* regulates the rhythmicity of immobility in a sex-specific pattern.

Few studies have investigated sex-specific differences in response to stress and depression-like behavior. One study did investigate the influence of sex on depression-like behavior during different phases of the circadian cycle [24]. Consistent with our results, they found that female rats had more immobility in the dark phase in comparison to male rats. However, they did not find a circadian phase effect in males [24]. There may be several reasons for this result, which differs from ours. The experiments examined different model systems (mice *vs.* rat). Additionally, the circadian rhythm of the rats was reset by reversing the light cycle, which may stress the animals, leading to potential effects on late behavioral testing. Finally, all behavioral testing of the rats was performed during a single time period, preventing analysis of the full range of circadian changes.

Our study reveals that there are distinct, sex-specific differences in the levels of immobility among the mouse strains deficient in core circadian genes. *Per1^Brdm1^* female mice show higher levels of immobility in comparison to males, while female *Per2* and *Clock* mutant mice show consistently lower levels of immobility in both day and night compared to males of the appropriate genotype. This finding may result partly from the complex crosstalk between the circadian CLOCK system and sex hormones. For example, the circadian genes, *mPer1*, *mPer2*, and *mClock* are also involved in the regulation of the female reproductive and estrous cycles, which may further influence behaviors [25,26]. Similarly, fluctuations in ovarian hormones have area-specific effects on the expression of *Per2* in the brain [27].

However, there are limitations in our study. The estrus cycle of the female mice was not considered. It had been reported that female rats in proestrous and estrous phases exhibit more immobility than animals in the diestrous phase [28]. Another study, however, indicate that the estrous cycle did not significantly modulate behavioral outcomes tested by FST [29]. In our study, female mice were grouped together in different estrous cycles, and mice in different estrous cycles were included in each group, which may balance the potential influences of the estrous cycle partly. Nevertheless, estrus cycles should be more carefully considered in our future studies. Another limitation in our study is we only tested immobility behavior induced by FST, the data need be further determined by another measure, such as the tail suspension test or learned helplessness.

In summary, our results highlight the differences in the circadian characteristics of immobility induced by FST in wild type, *Clock^Δ19^*, *Per1* (*Per1^Brdm1^*), and *Per2* (*Per2^Brdm1^*) mutant mice. All four genotypes showed sex-specific differences in the level of immobility, while sex-specific differences in circadian patterns only occurred in WT mice. This result indicates that there is no common behavioral profile associated with the disruption of individual core circadian genes, perhaps because of the complex crosstalk between the circadian CLOCK system and sex hormones. Our study supports the hypothesis that the disturbance of biological clocks contributes to depression and improves our understanding of the involvement of circadian genes in the regulation of mood disorders.

## 3. Materials and Methods

### 3.1. Subjects

The subjects were ten- to twelve-week-old *Clock^Δ19^*, *Per1* (*Per1^Brdm1^*) and *Per2* (*Per2^Brdm1^*) mutant mice and age- and sex-matched WT C57BL/6J control mice. All mutant mice were backcrossed on a C57BL6 genetic background for 20 generations. All animals were housed in a temperature (21 ± 2 °C) and humidity (50% ± 5%) controlled room with food and water freely available in the home cages. Before each experiment, all mice were kept for at least 6 weeks in a 12-h light/12-h dark cycle, with lights on at 7:00 a.m. (referred to as zeitgeber time point 0, ZT0) and lights off at 7:00 p.m. (referred to as ZT12). All animal procedures were performed in accordance with the National Institute of Health’s Guide for the Care and Use of Laboratory Animals, and all procedures were approved by Institutional Review Board (Protocol Number: A12031; May 2012), Institute of Psychology, Chinese Academy of Sciences.

### 3.2. Forced Swim Test

The forced swim test was performed as originally proposed by Porsolt *et al.* [30] and modified by Hirani *et al.* [31]. Two sessions were used in the present study. A “pre-test session” can prevent variations and help maintain consistency in the immobility time between different groups [31]. Briefly, mice were forced to swim for 15 min in an acrylic circular cylinder (35 cm in diameter) filled with 25 °C water to a depth of 30 cm. After pre-exposure, mice were placed in a separate cage, towel dried, and then returned to their home cage. Twenty-four hours later, they were submitted to a 5 min test session of forced swim, and the total time spent immobile was recorded. The test was performed during nighttime in the darkness. The mice were judged immobile when they ceased struggling and remained floating motionless in the water (without any vertical or horizontal movements), making only the movements necessary to keep their heads above the water level. Naïve mice were used to perform FST at each time point (*n* = 6–9 for each group at each time point).

### 3.3. Statistical Analyses

The circadian rhythms of dependent variables were generated using the single cosinor method to calculate the mesor (*i.e.*, the middle value of the fitted cosine that represents a rhythm adjusted mean), the amplitude (*i.e.*, half of the difference between the minimum and maximum of the fitted cosine function), and the standard error of their dispersions [32]. The duration of immobility of peak and nadir were compared by *t*-test. Sex-specific differences in the duration of immobility for each type of mouse were analyzed using two-way analysis of variance (ANOVA) followed by least significant difference (LSD) *post hoc* with the between sex factors (male or female) and the within subjects factor of test condition (Zeitgeber time). The level of statistical significance was set at *p* < 0.05.
